# A Review of Smartphone Apps Used for Toric Intraocular Lens Calculation and Alignment

**DOI:** 10.3390/vision6010013

**Published:** 2022-02-18

**Authors:** Yarrow Scantling-Birch, Hasan Naveed, Ritika Mukhija, Mayank A. Nanavaty

**Affiliations:** 1 Sussex Eye Hospital, University Hospitals Sussex NHS Foundation Trust, Brighton BN2 5BF, UK; yarrow.scantling-birch@nhs.net (Y.S.-B.); ritika.mukhija@nhs.net (R.M.); 2 Maidstone Hospital, Maidstone and Tunbridge Wells NHS Trust, Maidstone ME16 9QQ, UK; hasan.naveed@nhs.net; 3 Brighton and Sussex Medical School, University of Sussex, Falmer Campus, Brighton BN1 9PX, UK

**Keywords:** smartphone, technology, applications, toric, intraocular, lenses, refractive, surgery

## Abstract

Smartphone apps are becoming increasingly popular in ophthalmology, one specific area of their application being toric intraocular lens (IOL) surgery for astigmatism correction. Our objective was to identify, review and objectively score smartphone apps applicable to toric IOL calculation and/or axis alignment. This review was divided into three phases. A review was conducted on four major app databases (phase I): National Health Service (NHS) Apps Library, Google Play Store, Apple App Store and Amazon Appstore. A systematic literature review (phase II) was conducted to identify studies for included apps in phase I of our study. Keywords used in both searches included: “toric lens”, “toric IOL”, “refraction”, “astigmatism”, “ophthalmology”, “eye calculator”, “ophthalmology calculator” and “refractive calculator”. Included apps were objectively scored (phase III) by three independent reviewers using the mobile app rating scale (MARS), a validated tool that ranks the quality of mobile health apps using a calculated mean app quality (MAQ) score. Phase I of our study screened 2428 smartphone apps, of which six apps for toric IOL calculation and four apps for axis marking were eligible and were selected for quantitative analysis. Phase II of our study screened 477 studies from PubMed, Medline and Google Scholar. Three studies validating two apps (toriCAM, iToric Patwardhan) in a clinical setting as adjunct tools for preoperative axis marking were identified. Phase III ranked Toric Calculator for iPhone (Apple iOS, MAQ 4.13; average MAQ 3.34 ± 0.54) as the highest-scoring toric IOL calculator, and iToric Patwardhan (Android OS, MAQ 4.13; average MAQ 3.41 ± 0.44) was the highest-scoring axis marker in our study. Our review identified and objectively scored ten smartphone apps available for toric IOL surgery adjuncts. Toric Calculator for iPhone and iToric Patwardhan were the highest-scoring toric IOL calculator and axis marker, respectively. Current literature, though limited, suggests that axis marking smartphone apps can achieve similar levels of misalignment reduction when compared to digital systems.

## 1. Introduction

Toric intraocular lens (IOL) implantation is one of the most effective ways to correct corneal astigmatism during cataract surgery; however, its practical use relies on accurate calculation, selection and alignment of the toric IOL [1,2]. There are multiple steps involved in the preoperative planning for toric IOL implantation, including patient selection, accurate biometry, consistency with regular and symmetric corneal astigmatism on corneal topography and reference markings [3]. Intraoperatively, implanting the IOL at the correct axis is of utmost importance as deviations as small as 3 degrees from the intended axis can result in a clinically significant 10% loss in astigmatism correction, with a rotation of 30 degrees resulting in a 100% loss of astigmatic correction [1,4]. In an extensive retrospective review of 4949 eyes, the primary sources of error identified during toric IOL surgery included preoperative measurements of the cornea (27%), IOL misalignment (14.4%) and IOL tilt (11.3%) [5]. These errors, subsequently, may require further treatment in the form of IOL repositioning, exchange or laser refractive surgery [6]. In our experience, 72% of the eyes with preoperative astigmatism between 0.75 and 2.5 diopters (D) were within 0.5 D of residual manifest astigmatism and 63% of eyes had an astigmatic error of ≤0.5 D relative to the expected residual astigmatism at 12 months after toric IOL implantation [1].

The modern era has seen a proliferation of smartphone technology [7,8]. Up to 90% of healthcare professionals already use smartphones daily [9], and there are over 400,000 healthcare-related apps available online [10]. This culture shift and the development of specialised apps offer ophthalmic surgeons adjunctive tools to assist and check toric IOL calculations and alignment. Several reviews highlighted recommended apps for a general ophthalmology audience [9,11,12], and specific studies explored smartphone apps in refractive surgery [8,13,14,15,16]. However, there remains a paucity of reviews that summarise the available smartphone apps to aid toric IOL calculation and axis alignment, and much is unknown about the quality of these apps.

Given the factors above, our group aimed to identify and appraise smartphone apps designed to assist toric IOL calculation and axis alignment, perform a literature review on these identified apps and objectively score these apps using a validated mobile app rating scale.

## 2. Material and Methods

This review was conducted at the Sussex Eye Hospital, University Hospitals Sussex NHS Trust, Brighton, United Kingdom. No ethical approval was sought for our study, as per the Health Research Authority (HRA) Research Ethics Committee (REC) online tool. This study was conducted in three phases:

Phase 1. A review of all the available smartphone apps for toric IOL calculations and axis alignment.

Phase 2. A systematic literature review to identify studies for included apps identified in phase I.

Phase 3. Objective scoring of these apps using the validated mobile app rating scale (MARS) [17].

### 2.1. Phase I. A Review of All Available Smartphone Apps for Toric IOL Calculations and Axis Alignment

**Figure 1:**
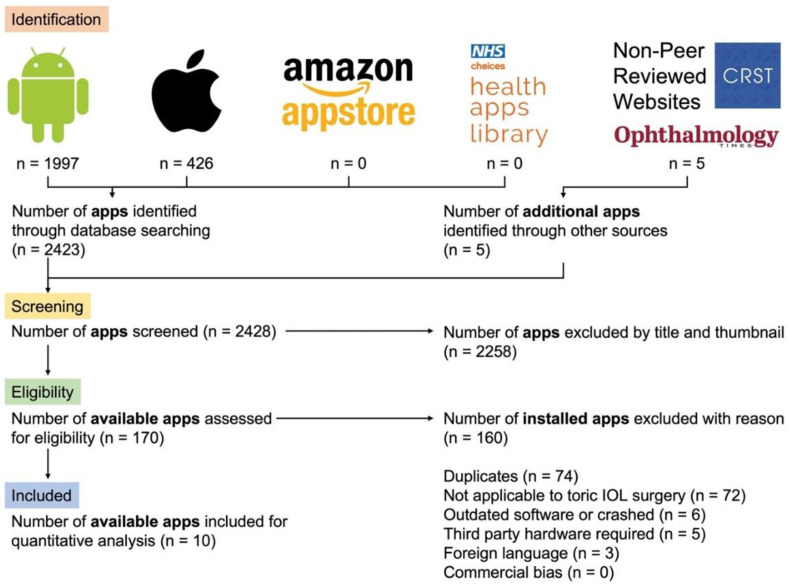
PRISMA flow diagram to highlight the systematic review of the smartphone app stores using keywords “toric lens”, “toric IOL”, “refraction”, “astigmatism”, “ophthalmology”, “eye calculator”, “ophthalmology calculator” and “refractive calculator” in NHS Apps Library [18], Google Play Store [19], Apple App Store [20], Amazon Appstore [21] and non-peer-review ophthalmology websites [22,23,24,25,26].

Healthcare provider stores [18], commercial app stores [19,20,21] and popular, non-peer-reviewed ophthalmology websites [22,23,24,25,26] were searched using the following keywords: “toric lens”, “toric IOL”, “refraction”, “astigmatism”, “ophthalmology”, “eye calculator”, “ophthalmology calculator” and “refractive calculator”. Inclusion and exclusion criteria were established for identifying smartphone apps (Table 1). The systematic app search was performed in March 2021 by two independent reviewers (Y.S.B., H.N.), and an agreement was reached on included apps following the Preferred Reporting Items for Systematic Reviews and Meta-Analyses (PRISMA) guidelines (Figure 1) [27]. During our systematic review of app stores, there was no function to remove duplicates between the major app databases. A limit of only first-page internet was applied to non-peer-reviewed websites. The screening was based on app title and thumbnail alone. Eligible apps were installed onto a supporting operating system (OS), tested briefly for functionality in a non-clinical setting by two reviewers (Y.S.B., H.N.) and then re-assessed against the same inclusion and exclusion criteria (Table 1). The included apps underwent quantitative analysis in study phase III using the mobile app rating scale (MARS) by three observers (Y.S.B, H.N., R.M.) [17]. Additional app metrics collected during the systematic search strategy included: app name, smartphone OS (Android, Apple), cost (British pound sterling, GBP), function (relating to toric IOL surgery), app rating (1–5 stars), number of reviews and the developer(s) (clinician, app developer, industry, other). Where necessary, a combination of the United Kingdom (UK) app store, the United States (US) app store and a third-party website for the app analytics [28] were used to derive additional app metrics.

### 2.2. Phase II. Systematic Review to Identify Studies That Included the Smartphone Apps Identified in Phase I

**Figure 2:**
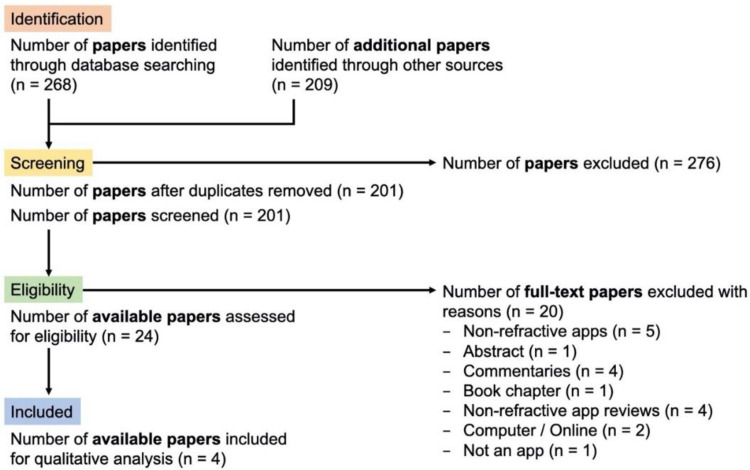
PRISMA flow diagram to highlight the systematic review of the literature using keywords [“toric lens” OR “astigmatism” OR “refraction”] AND [“calculator” OR “mobile application” OR “mHealth”] to search PubMed, Medline and Google Scholar. 477 papers were identified, 201 screened, 24 assessed in full text and 4 studies included.

A combination of keywords and MeSH terms relating to [“toric lens” OR “astigmatism” OR “refraction”] AND [“calculator” OR “mobile application” OR “mHealth”] were used to search PubMed, Medline and Google Scholar. The search was performed during February 2021 by two independent reviewers (Y.S.B., H.N.) and by PRISMA guidelines (Figure 2) [27]. All abstracts were reviewed, and potentially eligible articles for the apps identified in phase I were read in full. A final list of studies meeting the eligibility criteria was compared, and disagreements were resolved by discussion between the two authors. Articles, written in English, validating an app to assist toric IOL surgery or discussing the development of new apps for toric IOL surgery were included. Studies were excluded if they did not provide original data or relate to smartphone apps that assist toric IOL surgery. One reviewer (Y.S.B.) extracted the following data for each included study: authors, publication year, country of study, study design, setting, sample size, smartphone app used, the app’s purpose, controls and primary outcome.

### 2.3. Phase III. Objective Scoring of the Included Apps Using the Validated Mobile App Rating Scale (MARS)

Included apps from phase I were scored by three independent observers (Y.S.B., H.N., R.M.) using the mobile app rating scale (MARS) to assess the quality. The MARS was developed by Stoyanov et al. [17] and is a popular, validated tool that scores the quality of mobile health apps using a 5-point Likert scale (1—Inadequate, 2—Poor, 3—Acceptable, 4—Good, 5—Excellent) in five domains: engagement, functionality, aesthetics, information quality and subjective quality [29,30,31,32]. Each domain has ‘descriptive terms’ and ‘technical aspects’ to prompt the consideration of reviewers when scoring an app on the 5-point Likert scale. For example, the aesthetics domain asks the user to consider the graphics, layout and visual appeal (Table 2). The MARS employs objective domains (engagement, functionality, aesthetics and information quality) and a subjective domain (subjective quality). The subjective quality (SQ) can be scored separately, whilst the mean scores of engagements, functionality, aesthetics and information quality (IQ) combine to provide a mean app quality score (MAQ).

The included apps were coded remotely using an online survey (Qualtrics^XM^, Seattle, WA, USA) completed independently by all three observers in July 2021. Two ophthalmology trainees independently provided scores for each app (H.N. scored iOS apps on iPhone XR 14.7.1 iOS, and R.M. scored Android apps on Oneplus 6 10.3.12 OxygenOS). A third trainee (Y.S.B.) independently provided a second score for all included apps (both iOS and Android using the same devices). The mean of the two scores (derived by Y.S.B. and H.N. and Y.S.B. and R.M. for iOS and Android apps, respectively) was taken to represent each domain of the MARS. If clinical data were required to score an app (e.g., axis marking), the individual reviewers imported images (e.g., picture of an eye) or used mock patient data excluding any patient-identifying features. The MAQ of each app (highlighted in bold in Table 3) was calculated and used to rank apps from highest scoring to lowest scoring in the categories of toric IOL calculation (Table 3A) and axis markers (Table 3B). Correlations between the MAQ and other app metrics, including cost, subjective quality, app rating and number of reviews, were calculated to identify any positive relationships.

Data analysis was performed using Excel (Microsoft Corporation, Redmond, WA, USA) and GraphPad Prism software (GraphPad Software Incorporated, San Diego, CA, USA). Descriptive data are presented using simple statistics and tables. Continuous data are presented as means and standard deviation (SD). Categorical data are presented as counts and percentages (%). The distribution of outcomes was assessed. A non-parametric statistical test (Spearman’s rank correlation) was used to assess the strength of relationships between MAQ and specific app metrics (subjective quality, app rating, number of reviews and cost). A *p*-value =< 0.05 was deemed to be statistically significant.

## 3. Results

### 3.1. Phase I. A Review of All Available Smartphone Apps for Toric IOL Calculations and Axis Alignment

A total of 2428 smartphone apps were identified and screened during the systematic search strategy of four major app databases and additional, non-peer-reviewed online sources (Figure 1). Two thousand two hundred and fifty-eight smartphone apps were excluded based on title and thumbnail alone. One hundred and seventy apps were eligible for download and brief testing. Ten apps, including toric IOL calculation (*n* = 6) and axis alignment (*n* = 4), were selected for quantitative analysis using the MARS scoring (Table 2). All smartphone apps were downloadable from either the Google Play Store or Apple App Store. No app was multiplatform or downloadable from the Amazon Appstore or NHS Health Apps Library. There was an even split between smartphone apps available for different operating software, including five for Android (IOL calculation *n* = 3; axis marking *n* = 2) and iOS (IOL calculation *n* = 3; axis marking *n* = 2). Most apps were free to purchase, with the remaining costing between GBP 1.99 and GBP 4.99. All results are summarised in Table 3A,B.

### 3.2. Phase II. Systematic Review to Identify Studies That Included the Smartphone Apps Identified in Phase I

A total of 477 studies was identified in the systematic search strategy that either described, used or validated apps to aid toric IOL surgery (Figure 2). Two hundred and seventy-six papers were removed due to duplication, and 177 were excluded following abstract review. After reading the remaining 24 articles in full, a further 20 were excluded. A total of three articles, all about axis marking, were eligible for inclusion in this systematic review (Table 4). No studies validated toric calculators or other measures to aid toric IOL surgery.

Pallas et al. [13] used an Australian mock patient cohort (40 eyes) in a tertiary eye unit to demonstrate the value of smartphone-assisted reference marking as an adjunct to the traditional standards of slit-lamp and freehand marking. They established that the toriCAM smartphone app (axis marker) reduced the mean absolute error of measurement from 3.18° ± 2.22° to 1.28° ± 1.34° (*p* < 0.01), which translated into an average reduction in marking error of 59.8% for the entire cohort. These measurements were verified using an automated reference marking method (iTrace with Zaldivar Toric Caliper, Tracey Technologies, Houston, TX, USA).

In the same group in Western Australia, Lipsky et al. [15] used the toriCAM smartphone app (axis marker) as an adjunct tool to determine the true meridian of the reference mark intraoperatively in two groups (36 eyes each) undergoing different manual reference marking: Barrett dual-axis toric marker and Mendez gauge. The mean absolute alignment error was significantly lower in the group that employed both the toriCAM and the Barrett dual-axis toric marker (4.0 ± 2.9°) than in the toriCAM plus Mendez gauge group (8.4 ± 6.5°; *p* < 0.001). This translated into postoperative outcomes where the percentage of eyes achieving a postoperative manifest refraction cylinder of 0.50 D or less was significantly higher in the group using the toriCAM smartphone app and the Barrett dual-axis toric marker (29 versus 21, *p* < 0.05). 

Khatib et al. [8] examined an alternative, axis marking smartphone app (iToric Patwardhan). They compared it to the manual, freehand, slit-lamp method in a prospective study from Western India involving 42 eyes. The Verion Digital Marker (Alcon Laboratories, Fort Worth, TX, USA) was used as a gold standard to compare any errors between marking methods. They found that smartphone-aided marking using an axis marker had significantly lower angular deviations than manual marking methods (2.62° ± 2.40° versus 4.60° ± 2.96°; *p* <0.01), improving IOL placement accuracy overall.

### 3.3. Phase III. Objective Scoring of the Included Apps Using the Validated Mobile App Rating Scale (MARS)

Among the toric IOL calculation apps, the highest scoring was Toric Calculator for iPhone (Apple iOS, MAQ 4.13), followed by Eye Tools (Apple iOS, MAQ 3.88). The lowest scoring was Excellent Toric Calculator (Android OS, MAQ 2.50), with an average score of 3.34 ± 0.54 amongst the six apps. The highest-scoring objective domain of the MARS was functionality (3.75 ± 0.52), followed by information quality (3.50 ± 0.55). Interestingly, most toric IOL calculation apps were developed by industry, either alone (*n* = 2) or in collaboration with developers (*n* = 3), and only one was developed by an ophthalmic clinician. The average number of reviews per app was 5.2, ranging from none for Excellent Toric Calculator and Toric Calculator for iPhone to 17 for Biotech Calculators. Spearman’s rank correlation coefficients (r and *p* values) demonstrated no statistically significant correlation between MAQ and subjective quality (+0.63, *p* = 0.50), app rating (+0.40, *p* = 0.75), several reviews (+0.20, *p* = 0.92) and cost (+0.26, *p* > 0.99).

Amongst the axis alignment apps, the highest scoring was iToric Patwardhan (Android OS, MAQ 4.13), followed by Axis Assistant (Apple iOS, MAQ 3.38). The lowest scoring was Toric IOL Axis Marker (Android OS, MAQ 3.00), with an average of 3.41 ± 0.44 amongst the four apps. The highest-scoring objective domain of the MARS was functionality (3.88 ± 0.48), followed by a combined second of engagement (3.25 ± 0.65), aesthetics (3.25 ± 0.29) and information quality (3.25 ± 0.87). All axis marking apps were developed by ophthalmic clinicians, either independently (*n* = 3) or in collaboration with developers (*n* = 1). The average number of reviews per app was 11.5, ranging from 6 for Axis Assistant to 19 for iToric Patwardhan. Spearman’s rank correlation coefficients (r and *p* values) demonstrated a statistically significant positive correlation between MAQ and subjective quality (+0.96, *p* < 0.01). There was no statistically significant correlation between MAQ and app rating (+0.21, *p* = 0.83), number of reviews (−0.08, *p* = 0.86) and cost (+0.79, *p* = 0.07).

## 4. Discussion

Smartphone apps that assist with daily clinical practice are rapidly gaining popularity in medicine, especially in ophthalmology [9,11,12]. Toric IOL misalignment remains the second largest error that contributes to loss in astigmatic correction during refractive surgery [5]. Toric IOL implantation is one of the domains in ophthalmology where smartphone apps may help improve postoperative outcomes through the automation of IOL selection and alignment, reducing the need for subsequent revisions [6]. We identified six apps for toric IOL calculation and four for axis alignment. Two of these apps (toriCAM, iToric Patwardhand) were validated further in a clinical setting as adjunct tools for preoperative axis marking [8,13,15]. According to the MARS [17], Toric Calculator for iPhone was the highest-scoring toric IOL calculator, and iToric Patwardhan was the highest-scoring axis marker in our review.

In our study, Toric Calculator for iPhone (Apple iOS, MAQ 4.13) was one of the top-performing apps for toric IOL calculation and scored highly in all four objective domains: engagement 4.0, functionality 4.5, aesthetics 4.0 and information quality 4.0. This was due to the aesthetically pleasing user interface and added-value functions, including toric lens details, various commercial formulae, diagrams for toric IOL placement and patient data storage. Furthermore, the app gave helpful preoperative values regarding the axis of toric IOL placement, incision location (degrees) and the anticipated residual astigmatism (degrees). These traits were shared amongst the other top-performing toric calculators, including Eye Tools (Apple, MAQ 3.88) and Biotech Calculators (Android, MAQ 3.25). These apps had the ability to serve multiple functions, capacity for data storage, pleasing aesthetics and overall usability with minimal malfunctions. 

Apps that scored poorly, such as Excellent Toric Calculator (Android OS, MAQ 2.50), had poor navigation, lack of updates, associated software latency and aesthetically displeasing design on larger phone screens. This was consistent with previous work that shows apps’ appearance and corresponding icons impact popularity in app stores; specifically, apps with high colourfulness and brightness receive a more significant number of downloads [33]. Interestingly, there was no statistically significant positive correlation between our study’s MAQ scores and app ratings or the number of reviews. The latter two metrics dictate audience engagement and subjective opinions on apps. However, app store ratings and reviews can be highly subjective, not reflecting the genuine opinion of users and, in some cases, carrying significant biases: either individuals with extreme opinions or ‘paid likes’ from third-party industries invested in an app’s financial success [34].

Toric IOL calculators come close to allowing smartphone users to select toric IOLs by inputting manual keratometry measurements and applying different commercial formulae to calculate the power of toric IOL. All toric calculation apps used basic parameters, vis-à-vis, Flat K with axis, steep K with axis, surgically induced astigmatism (SIA) and incision axis for calculation of cylinder value of toric IOL and its placement; three apps (Eye Tools, EyeToric and Excellent Toric Calculator) further asked for axial length and A constant. Only one app (EyeToric) required the anterior chamber depth (ACD) and considered the effective lens position; however, none of the apps incorporated posterior corneal astigmatism. As three out of six apps did not require axial length or A constant measurements, no formula was used to calculate the spherical equivalent of IOL power, and the same had to be inputted manually. Out of the other three, EyeToric provided the option of using one of four formulae (SRKT, Haigis, Holladay, HofferQ). EyeTools provided the option of using one of three formulae (HofferQ, Holladay 1, theoretical SRKT). Excellent Toric Calculator did not provide this information.

However, these apps are not standalone and still rely on expensive, third-party commercial hardware to deliver corneal measurements while not integrating with the latest diagnostic technology [35]. It is essential to consider these limitations of smartphone app technology in toric IOL surgery, in addition to the inability to measure keratometry, define corneal astigmatism and intraoperative feedback. Furthermore, no studies were identified in our systematic review validating the use of toric IOL calculators and, hence, there is no clinical reflection of the calculating apps in terms of post-op residual astigmatism and visual acuity in refractive cataract surgery. Compared to commercial, online calculators, toric IOL calculators, overall, are more compatible with various toric IOLs, have less bias for specific lens manufacturing company and have the added advantage of being available on portable smartphone devices for any rapid calculations.

An important preoperative step in accurately aligning a toric IOL is marking the reference meridian (0, 180° or 90°, 270°), which is then used intraoperatively to place the IOL at the desired axis of placement. Axis marking techniques can be either manual or automated; the latter uses image-guided software and computer-based systems and has been shown to deliver on minor axis misalignment with better postoperative refractive outcomes compared to manual axis marking techniques in toric IOL surgery [36,37,38]. This includes additional advantages of enhancing workflow, improving intraoperative accuracy through limbal registration and wavefront aberrometry and high-quality video documentation of the surgical procedure [38]. According to Moore’s law [39], the latest smartphones can act as mini-computers and mimic some of the advantages provided by digital marking methods with increasingly sophisticated microprocessors, high-definition cameras and a growing library of third-party smartphone apps. 

Khatib et al. [8] validated the iToric Patwardhan axis marking app in an appropriately powered prospective study of 42 eyes undergoing toric IOL surgery and showed a statistically significant reduction in mean absolute angular deviation using the app versus manual marking by approximately 2° along the horizontal meridian. This is critical since a deviation of 3° from the intended axis can equate to a 10% loss in toricity and worse postoperative refractive outcomes [2]. In addition, they were able to demonstrate a robust statistical agreement (intraclass correlation coefficient 0.88) in deviation measurements between the iToric Patwardhan app and the Verion Digital Marker (Alcon Laboratories, Fort Worth, TX, USA), suggesting almost similar gains with free-to-use smartphone apps as opposed to a larger and more expensive, commercial instrument. Our study supports iToric Patwardhan app (Android OS, MAQ 4.13) as the top-performing axis marking app, which scored high in functionality (4.5), information quality (4.5) and engagement (4.0). This can be attributed to its simplicity, aesthetically pleasing user interface, functionality and smooth integration with inbuilt smartphone features. Compared to other apps, iToric Patwardhan was one of four apps developed and designed specifically by an ophthalmologist, suggesting that app development should be a collaboration between clinicians and developers to maximise information quality, clinical utility and overall app usability [40]. 

Other axis marking apps included Axis Assistant (Apple iOS, MAQ 3.38), toriCAM (Apple iOS, MAQ 3.13) and Toric IOL Axis Marker (Android OS, MAQ 3.00). These collectively scored less due to downfalls, including lack of software updates, poor alignment with larger smartphone screens, lack of historical case storage and the singular function as a horizontal meridian marker. All axis marking apps included in this study served to only mark the reference meridians (horizontal axis) and were dependent on second instruments, such as a Mendez ring for toric IOL alignment or optical biometry machines. Pallas et al. [13] validated the toriCAM app in a prospective study of 40 eyes (mock patient cohort). They found a clinically significant reduction in the mean absolute measurement error of 1.9° when used in adjunct with both manual methods (slit-lamp and freehand marking). Lipsky et al. integrated the toriCAM app with manual axis marking methods (Mendez gauge versus Barrett dual-axis toric marker). They found that the smartphone app could be an adjunct in reducing postoperative toric IOL alignment in both manual methods. 

All three studies validating axis marking smartphone apps [5,8,13] achieved similar levels of misalignment reduction to that of digital axis marking techniques from more sophisticated, computer-based systems, such as iTrace (Tracey Technologies, Houston, TX, USA) and Verion Digital Marker (Alcon Laboratories, Fort Worth, TX, USA) [38]. Depending on the study, the use of digital marking techniques achieved between 1.4° and 1.9° in the postoperative toric IOL misalignment reduction [36,37], suggesting that smartphone apps may have a crucial role in contributing to the marginal gains in error reduction in the preoperative setting and subsequent improvements in refractive outcomes. However, the limitations of smartphone technology need to be acknowledged. This includes the lack of intraoperative feedback using more sophisticated techniques, such as image-guided wavefront aberrometry, and ethical concerns relating to confidential patient data on cloud-based storage.

One of the strengths of this study was the use of the well-established MARS, an objective scoring tool previously validated in numerous clinical studies [17,29,30,31,32]. In addition, a thorough novel systematic search strategy was employed for smartphone apps, covering the four major app databases, the two most popular smartphone OSs (Android and Apple) and adhering to PRISMA guidelines [27]. However, there were some limitations to our study. We only included smartphone apps, ignoring apps available on web-based or smart tablets. Secondly, one app was included despite industry bias (Biotech Calculators); however, it had the ability to accept lens values with similar toricity from other lens companies. Thirdly, smartphone apps greater than GBP 20 were excluded. This included an app that warrants review in future studies (Eye Pro by EB Eye Limited, toric calculator and axis marker, iOS, GBP 129.99). Fourthly, the medico-legal and ethical challenges posed by using apps in a clinical environment remains unknown. Many of the third-party app developers excuse themselves of any clinical liabilities, acknowledge issues relating to confidentiality and recommend the use of these apps in the hands of a professional. Fifthly, there are few clinical validating data to support the clinical accuracy of the toric IOL calculation apps. Some calculators still use a fixed corneal–IOL astigmatism correction ratio, which might lead to toric under- or overcorrection. Finally, there is a rapid turnover of smartphone apps; some apps present in this review may have already been removed from the market, whilst newer apps may have been released since this article. For example, Excellent Toric Calculator (app identified in March 2021) was no longer available on the Android app market at the time of study dissemination (December 2021).

## 5. Conclusions

The digital revolution is changing the landscape of toric IOL surgery as more smartphone apps can be used as an adjunct tool for IOL selection and alignment. Our review identified six toric IOL calculators and four axis marking apps available to cataract surgeons through a systematic review of the significant app databases and available literature. Using an objective scoring tool, we identified Toric Calculator for iPhone (Apple iOS, MAQ 4.13) as the highest-scoring toric IOL calculator and iToric Patwardhan (Android OS, MAQ 4.13) as the highest-scoring axis marker in our study. Future work needs to further validate these smartphone apps in clinical practice to establish a role in routine toric IOL surgery.

## Figures and Tables

**Table 1:** Inclusion and exclusion criteria for our systematic app search strategy.

Inclusion Criteria:
App is applicable to toric IOL selection, planning and surgery (e.g., keratometry, toric IOL selection, calculations, reference marking) Available for use and purchase by users based in the United Kingdom (UK) Available in the English language Available on popular smartphone operating software (OS) (e.g., Google Android or Apple iOS) Installation and use possible on the latest, up-to-date smartphone OS (Android OS 10+ and Apple iOS 14+) Run independently without additional requirements Free or purchasable within a budget of GBP 20 (£20)
**Exclusion Criteria:**
Apps that were designed for other purposes and not relating to toric IOL surgery(e.g., optometry, eye health, referrals, education, dictionary) Apps that were glitched or crashed after installation and use Apps that only ran on outdated smartphone OS (e.g., Blackberry, Windows) Apps that only ran on other smart devices or smart tablets Apps that had significant commercial bias or influence from lens companies (e.g., paid advertisement, mandatory registration with company) Apps that required third-party software or hardware (e.g., camera attachments, headsets)

**Table 2:** Five domains of the mobile app rating scale (MARS) and the associated ‘descriptor terms’ to prompt scoring on a 5-point Likert scale. There are four objective domains (engagement, functionality, aesthetics, information quality) and one subjective domain (subjective quality). Modified from Stoyanov et al. [17].

Engagement	Entertainment, Interest, Customisation, Interactivity, Target Group
Functionality	Performance, ease of use, navigation, gestural design
Aesthetics	Layout, graphics, visual appeal (how good does the app look?)
Information quality	Accuracy of app description, goals, quality of information, visual information, credibility, evidence base
Subjective quality	Would you recommend this app? How many times do you think you would use this app? Would you pay for this app? What is your overall star rating of the app?
Mean app quality (MAQ)	The MAQ can be calculated using the mean scores of engagement, functionality, aesthetics and information quality from the objective domains above.

**Table 3:** Summary of toric IOL calculation apps (*n* = 6) and summary of axis marking apps (*n* = 4).

A. Summary of Toric IOL Calculation Apps (*n* = 6).												
Rank	App Name	Operating System (OS)	Cost (GBP)	Engagement	Functionality	Aesthetics	Information Quality	Subjective Quality	Mean App Quality Score (MAQ)	App Rating ^1^ (1–5)	Reviews ^2^ (*n*)	Developer(s)
1	Toric Calculator for iPhone	iOS	3.99	4.0	4.5	4.0	4.0	4.0	**4.13**	-	0	App Developers and Industry
2	Eye Tools	iOS	4.99	4.0	4.0	3.5	4.0	3.5	**3.88**	4.0	1	App Developers and Industry
3	Biotech Calculators	Android	0.00	2.5	3.5	3.5	3.5	3.0	**3.25**	4.7	17	Industry
4	Axis Toric Calculator	Android	0.00	2.5	4.0	2.5	3.5	3.0	**3.13**	4.6	8	Industry
5	EyeToric	iOS	0.00	3.0	3.5	2.5	3.5	3.0	**3.13**	3.2	5	Ophthalmologist
6	Excellent Toric Calculator	Android	0.00	2.0	3.0	2.5	2.5	2.0	**2.50**	-	0	App Developers and Industry
**B. Summary of Axis Marking Apps (*n* = 4).**												
**Rank**	**App Name**	**Operating** **System (OS)**	**Cost (GBP)**	**Engagement**	**Functionality**	**Aesthetics**	**Information Quality**	**Subjective Quality**	**Mean App Quality Score (MAQ)**	**App Rating ^1^ (1–5)**	**Reviews ^2^ (*n*)**	**Developer(s)**
1	iToric Patwardhan	Android	0.00	4.0	4.5	3.5	4.5	4.5	**4.13**	4.7	19	Ophthalmologist
2	Axis Assistant	iOS	1.99	3.5	4.0	3.5	2.5	2.5	**3.38**	3.0	6	Ophthalmologist
3	toriCAM	iOS	0.00	3.0	3.5	3.0	3.0	3.0	**3.13**	4.3	7	Ophthalmologist
4	Toric IOL Axis Marker	Android	0.00	2.5	3.5	3.0	3.0	2.5	**3.00**	3.4	14	App Developers and Clinician

^1^ The Google Play Store for Android apps has ratings and reviews shared on an international platform. The Apple Store is country specific and, in this instance, ratings and reviews from the US store were used for Apple apps. This was due to lack of engagement from the UK store. ^2^ Certain apps had not acquired enough reviews, hence have been labelled as 0.

**Table 4:** Summary of included studies examining existing smartphone apps included in this study [8,13,15].

#	Authors (Year)	Country	Type of Study	Study Setting	Sample Size (No. of Patients)	Smartphone App	Purpose of App	Control(s)	Primary Outcome
1	Pallas et al. (2018)	Australia	Prospective randomised	Tertiary teaching hospital	22	toriCAM	Axis marking for the true reference meridian	Freehand marking vs. slit-lamp-assisted marking	Preoperative reference marking for toric IOL surgery
2	Lipsky et al. (2019)	Australia	Retrospective case series	Tertiary teaching and community hospital	56	toriCAM	Axis marking for the true reference meridian	Barrett dual-axis toric marker vs. Mendez gauge	Postoperative alignment error in toric IOL at 1 month
3	Khatib et al. (2020)	India	Prospective observational	Tertiary Eye Hospital	36	iToric Patwardhan	Axis marking	Manual marking vs. smartphone-assisted marking	Preoperative reference marking for toric IOL surgery

## Data Availability

Not applicable.
